# Spreading good news

**DOI:** 10.7554/eLife.07108

**Published:** 2015-04-02

**Authors:** Jeffrey A Fawcett, Hideki Innan

**Affiliations:** SOKENDAI (The Graduate University for Advanced Studies), Hayama, Japan; SOKENDAI (The Graduate University for Advanced Studies), Hayama, Japaninnanhk@soken.ac.jp

**Keywords:** *Drosophila miranda*, transposable elements, non-allelic gene conversion, regulatory networks, other

## Abstract

Gene conversion has a central role in the evolutionary fine-tuning of regulatory elements in the fruit fly *Drosophila miranda*.

**Related research article** Ellison CE, Bachtrog D. 2015. Non-allelic gene conversion enables rapid evolutionary change at multiple regulatory sites encoded by transposable elements. *eLife* **4**:e05899. doi: 10.7554/eLife.05899

**Image** Beneficial mutations can spread quickly via gene conversion to multiple locations in a genome

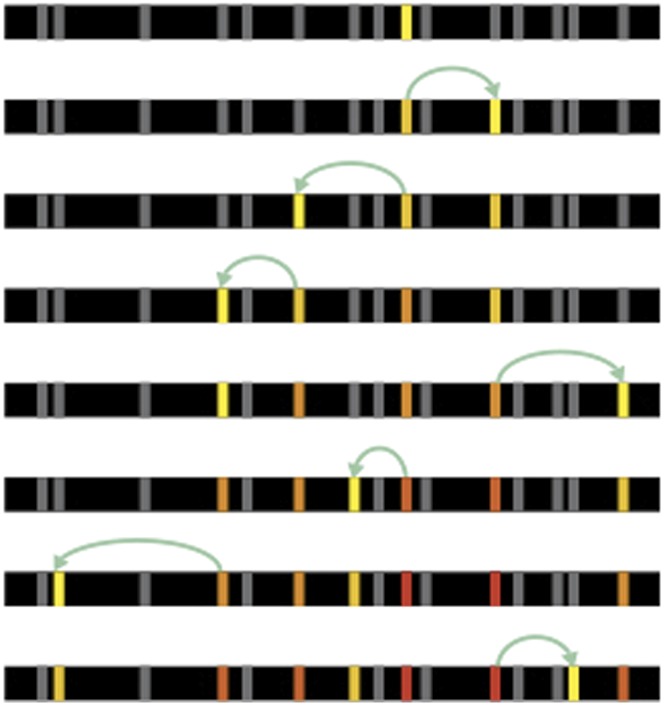

Natural selection means that an individual with a beneficial mutation is more likely to pass on its genes to its offspring. This ‘vertical’ process means that, eventually, the entire population can share the same beneficial mutation at the same location in the genome. However, beneficial mutations can also spread ‘horizontally’ so that they are shared between two (or more) locations in the genome of an individual (Figure 1). There are only a few ways that this horizontal movement can happen in animals and other eukaryotes. One way involves genetic parasites—called transposable elements—that ‘cut’ or ‘copy’ themselves from one location and then ‘paste’ themselves elsewhere in the genome. Gene conversion is another mechanism whereby one region of DNA is copied and used to replace a different, but highly similar, stretch of DNA. Now, in *eLife*, Christopher Ellison and Doris Bachtrog—both from the University of California, Berkeley—report that a beneficial mutation has spread via these two mechanisms to multiple sites on the X chromosome of the fruit fly *Drosophila miranda* (Ellison and Bachtrog, 2015).

**Figure 1.**
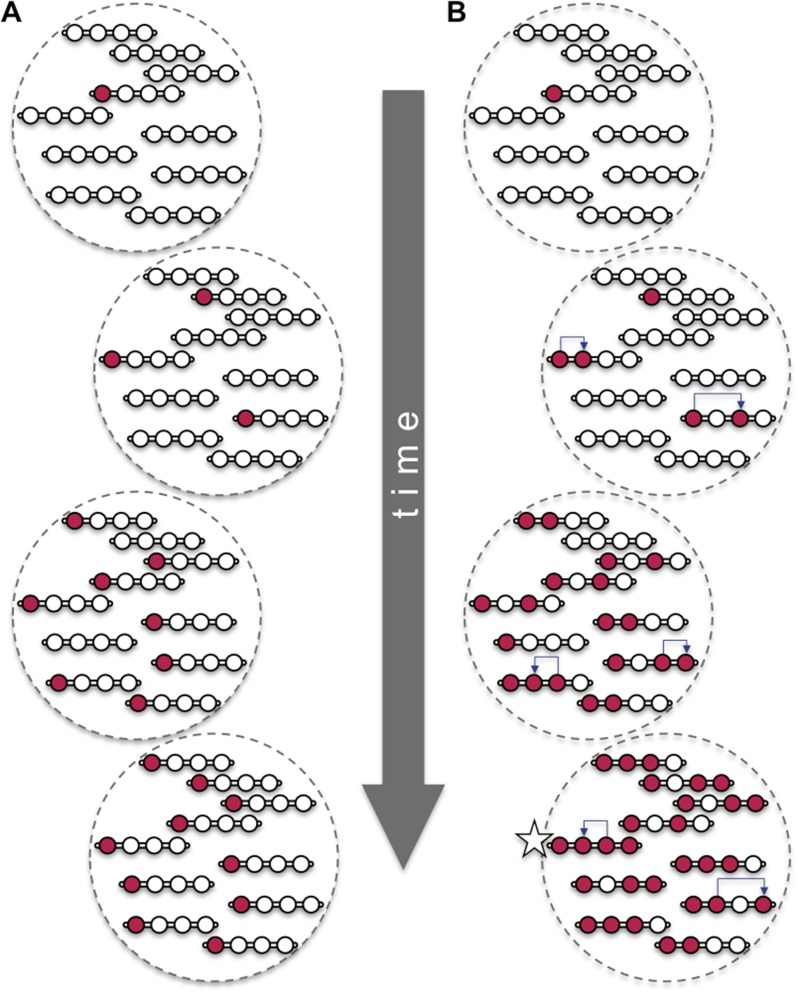
Beneficial mutations can spread through a population both ‘vertically’ and ‘horizontally’. Consider a hypothetical population with 10 individuals, each with a single chromosome that has four sites (open circles). (**A**) When beneficial mutations (red circles) are only passed ‘vertically’ between generations, the best possible outcome (shown in bottom dashed circle) is for all the individuals in the population to eventually have the beneficial mutation at the site where it appeared (i.e., the leftmost of the four circles). (**B**) However, when horizontal events (blue arrows) are involved, it is possible that an individual (marked with a star) can have the beneficial mutation at all four sites. Over time, the ‘vertical’ spread and natural selection can make it possible for all the individuals in the population to have the beneficial mutation at all four sites (not shown). These horizontal events could be the movement of a transposable element or, as Ellison and Bachtrog report, gene conversion.

Humans and many other animals have sex chromosomes. Human females are XX and human males are XY; this means that females have two copies of every gene on the X chromosome, but males only have one copy. Many sex chromosomes have a so-called ‘dosage compensation system’ to counteract this imbalance, which makes sure that males and females get an equal dose of the molecules made from the genes on the sex chromosomes.

A dosage compensation system is not something every sex chromosome has from its beginning. Instead, it has to be established through the course of evolution after the sex chromosome has formed. This process appears to be currently ongoing in *Drosophila miranda*, which developed a new X chromosome, called neo-X, about 1.5 million years ago, and provides an excellent opportunity to study how dosage compensation gets established (Ellison and Bachtrog, 2013).

In fruit flies, dosage compensation relies on a male-specific molecular complex that binds to a short sequence of DNA found multiple times across the X chromosome (Conrad and Akhtar, 2011). This complex then acts to increase the expression of nearby genes, which ensures that males receive a full dose of the molecules made from these genes. Thus, the most recently formed neo-X chromosome of *Drosophila miranda* needed to somehow develop sequence motifs that can also recruit this molecular complex.

Ellison and Bachtrog previously showed that such sequences are provided, in part, by a family of transposable elements that had recently copied itself across the neo-X chromosome (Ellison and Bachtrog, 2013). In fact, the binding motifs of the older X chromosome were also derived from transposable elements, and nature often seems to look to transposable elements when facing similar challenges. A number of studies have shown that transposable elements often serve as sources of ready-to-use binding motifs that can help regulate the expression of networks of genes (Cowley and Oakey, 2013; Sundaram et al., 2014). However, nature does not always get it right first time. Instead, the first attempt can be more of a ‘quick and dirty’ solution that is subject to further improvement over the course of evolution. Ellison and Bachtrog had previously found that the binding motifs derived from transposable elements on the neo-X chromosome are suboptimal (Ellison and Bachtrog, 2013). This led them to hypothesize that ‘fine-tuning’ of the process might be going on.

But, how could this fine-tuning be achieved? We can imagine that a beneficial mutation that allows the dosage compensation complex to bind more strongly might occur in one copy of the transposable element family. Would the only solution be for this transposable element to copy and paste itself across the neo-X again? In other words, would the entire dosage compensation network have to be rebuilt from scratch each time any copy acquires a better mutation?

Fortunately, it appears that ‘no’ is the answer to this question. Instead, nature seems to have a far more elegant solution that involves the spread of beneficial mutations via gene conversion. Ellison and Bachtrog identified two mutations in the transposable element that make it better at recruiting the molecular complex that carries out dosage compensation. They then showed that these two mutations are spreading across the different copies of the transposable element-derived regulatory element on the neo-X by gene conversion. Of course, gene conversion can work in both directions; that is to say, the process can spread a mutation or reverse it. However, because the mutations found by Ellison and Bachtrog are beneficial, natural selection tend to favor individuals where the mutation has spread over those where it has been reversed.

Gene conversion provides three major advantages in spreading beneficial mutations. First, mutations that are closer together, as is the case with the two mutations identified by Ellison and Bachtrog, can be transferred simultaneously. Second, it allows genetic variation to migrate across multiple sites in the genome, which, in turn, actually makes natural selection more efficient (Mano and Innan, 2008). Third, beneficial mutations present in different copies, or lineages, can be brought together. This may be analogous to the role of sexual recombination, which is considered as one of the major reasons why we have two sexes.

Although Ellison and Bachtrog only focused on the two mutations in this study, other beneficial mutations might also be present, or might arise in the future. It is likely that the fine-tuning of the dosage compensation system will continue by sharing such mutations across the entire family by gene conversion. It should be interesting to investigate whether such fine-tuning has occurred throughout the evolution of other regulatory networks.
